# Neuroimaging Biomarkers of mTOR Inhibition on Vascular and Metabolic Functions in Aging Brain and Alzheimer’s Disease

**DOI:** 10.3389/fnagi.2018.00225

**Published:** 2018-07-26

**Authors:** Jennifer Lee, Lucille M. Yanckello, David Ma, Jared D. Hoffman, Ishita Parikh, Scott Thalman, Bjoern Bauer, Anika M. S. Hartz, Fahmeed Hyder, Ai-Ling Lin

**Affiliations:** ^1^Sanders-Brown Center on Aging, University of Kentucky, Lexington, KY, United States; ^2^Department of Pharmacology and Nutritional Science, University of Kentucky, Lexington, KY, United States; ^3^F. Joseph Halcomb III, M.D. Department of Biomedical Engineering, University of Kentucky, Lexington, KY, United States; ^4^Department of Pharmaceutical Sciences, University of Kentucky, Lexington, KY, United States; ^5^Departments of Radiology and Biomedical Engineering, Magnetic Resonance Research Center, Yale University, New Haven, CT, United States

**Keywords:** mechanistic target of rapamycin (mTOR), rapamycin, caloric restriction, ketogentic diet, MRI, PET, Aging, Alzheimer’s disease

## Abstract

The mechanistic target of rapamycin (mTOR) is a nutrient sensor of eukaryotic cells. Inhibition of mechanistic mTOR signaling can increase life and health span in various species via interventions that include rapamycin and caloric restriction (CR). In the central nervous system, mTOR inhibition demonstrates neuroprotective patterns in aging and Alzheimer’s disease (AD) by preserving mitochondrial function and reducing amyloid beta retention. However, the effects of mTOR inhibition for *in vivo* brain physiology remain largely unknown. Here, we review recent findings of *in vivo* metabolic and vascular measures using non-invasive, multimodal neuroimaging methods in rodent models for brain aging and AD. Specifically, we focus on pharmacological treatment (e.g., rapamycin) for restoring brain functions in animals modeling human AD; nutritional interventions (e.g., CR and ketogenic diet) for enhancing brain vascular and metabolic functions in rodents at young age (5–6 months of age) and preserving those functions in aging (18–20 months of age). Various magnetic resonance (MR) methods [i.e., imaging (MRI), angiography (MRA), and spectroscopy (MRS)], confocal microscopic imaging, and positron emission tomography (PET) provided *in vivo* metabolic and vascular measures. We also discuss the translational potential of mTOR interventions. Since PET and various MR neuroimaging methods, as well as the different interventions (e.g., rapamycin, CR, and ketogenic diet) are also available for humans, these findings may have tremendous implications in future clinical trials of neurological disorders in aging populations.

## Introduction

The mechanistic target of rapamycin (mTOR) is a nutrient sensor that mediates the responses to energy status and growth factor in eukaryotic cells (Laplante and Sabatini, 2009). Discovered by three groups in 1994, mTOR is a particular protein bound by rapamycin (Brown et al., 1994; Cafferkey et al., 1994; Sabatini et al., 1994). mTOR activity can be inhibited by both rapamycin and nutritional signaling, such as caloric restriction (CR) (Perluigi et al., 2015). Inhibition of mTOR can switch cellular response from reproduction/growth to somatic maintenance, with decreased protein synthesis and cell growth, and increased autophagy in animal models (Harrison et al., 2009; Stanfel et al., 2009). As such, mTOR inhibition has shown to increase resistance to stresses resulting in lifespan extension in various mammalian species, and being considered central to the regulation of both aging and age-related diseases (Johnson et al., 2013).

In the central nervous system, mTOR inhibition has been shown to prevent neurodegeneration and protect brain functions in aging. Notably, rapamycin reduces amyloid-beta (Aβ) plaques and neurofibrillary tau tangles and improves cognitive functions in mice that model human Alzheimer’s disease (AD) (Spilman et al., 2010; Majumder et al., 2011). Similarly, CR (without malnutrition) is able to alleviate AD-like pathology (Lee et al., 2000, 2002; Thrasivoulou et al., 2006). In addition, CR protects mitochondrial function (the powerhouse in the cells), maintains glucose homeostasis, and reduces oxidative stress – all phenotypes of aging (Park et al., 2005; Duan and Ross, 2010; Perluigi et al., 2015). Thus, CR (reduced caloric and glucose intake) shifts metabolism toward ketone body utilization (Guo et al., 2015; Lin et al., 2015). Elevated ketone body metabolism or the administration of the ketogenic diet (KD) is also evident to be neuroprotective against AD, aging, epilepsy, brain injury, and neurodegeneration (Van der Auwera et al., 2005; Yang et al., 2017). However, biochemical and molecular experiments may limit mTOR-related research to *in vitro* or *ex vivo* cell culture or animal models. Such findings may be incapable of being completely translated and applied to humans.

Powerful brain imaging tools have been refined to visualize changes in brain function *in vivo* over time (Lavina, 2016; Hyder and Rothman, 2017). In particular, functional imaging can be used to determine changes in physiology before AD-like pathology appears and before the onset of cognitive impairment. Brain vascular and metabolic dysfunction plays a critical role in driving neurodegeneration and dementia (Reiman et al., 2001, 2004, 2005; Thambisetty et al., 2010; Fleisher et al., 2013). We have recently demonstrated that early detection of these physiological changes and identification of effective interventions using imaging would be critical to potentially slow down brain aging and prevent AD. **Table 1** summarizes the imaging techniques used in the studies we review, ranging from various magnetic resonance imaging (MRI) and spectroscopy (MRS) methods to positron emission tomography (PET) to confocal microscopic imaging, where the last method is primarily for preclinical research. To assess vascular functions, we used MRI-based arterial spin labeling (ASL), which measures quantitative cerebral blood flow (CBF) values by utilizing arterial blood water as an endogenous tracer. We also determined vascular density with magnetic resonance angiography (MRA) and blood-brain barrier (BBB) P-glycoprotein transport activity with live-cell imaging confocal microscopy. To assess metabolic functions, we used well-established PET protocols and proton magnetic resonance spectroscopy (^1^H-MRS) to quantify glucose uptake and brain metabolites, respectively (Lin et al., 2012; Lin and Rothman, 2014). We have also included the novel MRS techniques of ^1^H[^13^C] proton-observed-carbon-edited (POCE) to determine neurotransmission rate and mitochondrial oxidative metabolism in the aging brain.

**Table 1:** List of discussed neuroimaging methods.

Modality	Methods	Measurements	Applications
Magnetic resonance imaging (MRI) and spectroscopy (MRS)	ASL	Cerebral blood flow	Humans and animals
	MRA	Vascular density	
	^1^H-MRS	A variety of essential brain metabolites	
	POCE	Mitochondrial function; Neurotransmission rate; neuronal and glial activities	
Positron emission tomography (PET)	^18^FDG	Cerebral glucose metabolism	
Confocal Microscopy	Live-cell imaging	Blood brain barrier P-gp transport activity	Mainly in animals

*ASL, arterial spin labeling; MRA, magnetic resonance angiography; ^1^H-MRS, proton magnetic resonance spectroscopy; POCE, ^1^H[^13^C] proton-observed-carbon-edited MRS; ^18^FDG, fluorine-18 (^18^F)-labeled 2-fluoro-2-deoxy-d-glucose.*

In this review, we will discuss our neuroimaging findings on mTOR inhibition in the aging and AD brain. First, we will address the effectiveness of rapamycin in reducing AD-like pathology by restoring cerebrovascular functions in mice. Second, we will address our recent findings on CR and KD in enhancing brain vascular functions and shifting metabolism in young healthy mice. Third, we will provide evidence that CR preserves brain metabolic and vascular functions in aging in both mice and rats. Finally, we will discuss the translational potential of mTOR-related interventions in future human studies.

## Rapamycin Restores Brain Vascular and Metabolic Functions in Mice Modeling Human Alzheimer’s Disease

Rapamycin was discovered in 1970s from soil samples in Easter Island Rapa Nui (Sehgal et al., 1975); thus, the compound was named rapamycin (also known as sirolimus) after its place of origin (Johnson et al., 2013). It was discovered in 1988 that rapamycin contained immunosuppressive properties (Camardo, 2003). This finding led to the FDA’s approval of rapamycin in 1999 as an immunosuppressant preventative of the rejection in organs transplant patients (Camardo, 2003). Over the past two decades, rapamycin or its analogs have been widely used in the clinic and their toxicity profiles have been well characterized (Soefje et al., 2011).

Preclinical studies have been conducted to analyze the potential effectiveness of rapamycin to treat AD (Caccamo et al., 2010; Spilman et al., 2010; Majumder et al., 2011). In a recent study (Lin et al., 2017a), we focused on the effects of rapamycin in presymptomatic mice carrying the human apolipoprotein 𝜀4 (APOE4) allele, given that APOE4 is the most significant genetic risk factor for AD (Liu et al., 2013). Neuroimaging studies in humans have shown that cognitively normal APOE4 carriers develop vascular and metabolic deficits decades before the aggregation of Aβ and tau tangles (Reiman et al., 2001, 2004, 2005; Thambisetty et al., 2010; Fleisher et al., 2013). In particular, researchers conducting PET studies found that cognitively normal carriers of the APOE4 allele have abnormally low cerebral metabolic rates of glucose (CMRglc) in similar brain regions as patients diagnosed with AD (Reiman et al., 2001, 2004, 2005; Thambisetty et al., 2010; Fleisher et al., 2013). This metabolic abnormality was observed both in late-middle-aged (40–60 years of age) and young (20–39 years of age) carriers, who have normal memory and cognitive ability and are without Aβ or tau pathology. These PET findings suggest that APOE4 carriers develop functional brain abnormalities several decades prior to the potential onset of dementia. Longitudinal research using MRI has displayed that CBF is reduced in an accelerated manner in similar brain regions (e.g., frontal, parietal, and temporal cortices) in cognitively healthy APOE4 carriers (Thambisetty et al., 2010). The APOE4-related neurovascular risk is strongly correlated with an accelerated decline in verbal memory, language capability, attention, and visual/spatial abilities in midlife (Bangen et al., 2013).

A similar situation is seen in transgenic mice that express the human APOE4 isoform that is driven by the human glial fibrillary acidic protein promoter. Young, presymptomatic APOE4 mice have significantly lower CMRglc and CBF, as well as increased BBB leakage compared to the wild-type (WT) non-APOE4 mice (Bell et al., 2012; Lin et al., 2017a). Treating asymptomatic female APOE4 mice with rapamycin for 1 month resulted in a significant increase in CBF compared to the non-treated littermates. After 6 months of treatment, we found that rapamycin-treated APOE4 mice had normal CBF that was comparable to that of the sex- and age-matched WT mice. Similarly, rapamycin-treated mice also had lower BBB leakage. Furthermore, we found that BBB leakage could potentially be blocked by inhibiting cyclophilin A-dependent proinflammatory pathways with rapamycin (Bell et al., 2012). In addition, CMRglc was also restored to WT level as observed in the rapamycin-treated APOE4 mice (Lin et al., 2017a).

In another study with hAPP(J20) mice (a mouse model of human AD) that already developed significant Aβ pathology and cognitive decline, we found that rapamycin was also effective in restoring neurovascular function. Symptomatic hAPP(J20) 11 month old mice treated with rapamycin for 16 weeks had restored CBF to the level of WT mice (Lin et al., 2013). In addition, rapamycin restored vascular density, determined by MR angiography, in the brains of hAPP(J20) mice. The restored vascular integrity was highly correlated with reduced Aβ, cerebral amyloid angiopathy (CAA), and microhemorrhages in treated hAPP(J20) mice. These findings were consistent with the literature showing that rapamycin can reduce Aβ (Liu et al., 2017). In this study, we also identified that mTOR inhibition activates endothelial nitric oxide synthase (eNOS), and thus, released nitric oxide (NO), a vasodilator (Cheng et al., 2008; Lin et al., 2013). Therefore, rapamycin activating eNOS may be critical for restoring CBF in hAPP(J20) mice. In addition to restored cerebrovascular function and reduced AD-like pathology, hAPP(J20) mice also had improved memory and learning performance after 16 weeks of rapamycin treatment (Lin et al., 2013). Collectively, data generated from the two imaging studies show that rapamycin can potentially prevent AD phenotypes in APOE4 mice and reverse the effects of AD in hAPP(J20) transgenic mice (Richardson et al., 2015).

## Caloric Restriction and Ketogenic Diet Enhance Brain Vascular Functions and Shift Metabolism in Young Mice

In the early 1930s, Clive McCay demonstrated that restricting calorie intake without malnutrition can prolong both the mean and maximal lifespan in rats when compared to animals on *ad libitum* diet (AL; free eating) (McCay et al., 1989; Park, 2010). Since then, CR is perhaps the most studied anti-aging manipulation within a broad range of species (Colman et al., 2009; Choi et al., 2011; Rahat et al., 2011). This is further supported by other studies that display lower incidences of age-related neurodegenerative disorders found in animals treated with CR (Park et al., 2005; Duan and Ross, 2010).

Recently, our efforts have been focused on understanding how CR impacts brain function in the early stage. In particular, we would like to know how brain vascular and metabolic functions might be impacted with CR in young mice. We imaged mice at 5–6 months of age, either on 40% CR diet or AL, and found that CR significantly enhanced CBF (>20%) both globally and in the hippocampus, compared to their AL littermates (Parikh et al., 2016). The increase in CBF was associated with reduced mTOR and increased eNOS levels that were similar to what we observed with rapamycin. In addition, CR-fed mice had significantly increased P-glycoprotein (P-gp) transport activity levels at the BBB, which facilitates clearance of Aβ out of the brain. These findings are consistent with literature showing that CR reduces AD-like pathology and the onset of cognitive impairment (Mouton et al., 2009; Schafer et al., 2015).

We used ^1^H-MRS to determine energy metabolites in the hippocampus (Guo et al., 2015). We observed that CR mice displayed significantly increased levels of total creatine (tCr), a high-energy substrate, in comparison to AL mice. Given that tCr is the sum of creatine and phosphocreatine, we posited that CR increases adenosine triphosphate (ATP) production in young CR mice since phosphocreatine acts in a central role as an intracellular buffer during ATP production in mitochondria. This finding is consistent with literature that CR enhances ATP production by activating AMP-activated protein kinase (AMPK) and sirtuins pathways, which in turn suppresses the mTOR pathway (Blagosklonny, 2010). It has been found that the level of glucose regulates the AMPK pathway. With low levels of glucose and metabolic stress that accompany CR, there is a depletion of energy (low ATP: AMP ratio), which in turn activates AMPK (Salt et al., 1998; Hardie, 2014). AMPK, when activated, can be seen as an indicator of cellular energy status, turning on catabolic pathways that generate ATP while inhibiting cellular processes that consume ATP such as the mTOR pathway.

We also found significantly elevated levels of taurine in the CR mice when compared to the AL mice. Since taurine is correlated with neuromodulation, higher levels imply that young CR mice might have augmented excitability compared to the age-matched AL mice. Interestingly, both globally and in the hippocampus and frontal cortex (regions related to cognitive functions), CR mice displayed significantly reduced brain glucose uptake as determined by PET imaging (Guo et al., 2015). Our imaging findings are consistent with Western blot data showing that CR reduces glucose transporter 1 (GLUT-1) in brain capillaries of the mice (Parikh et al., 2016). As a result, we found a mismatch of CBF-CMRglc coupling induced by CR, opposite to tight coupling of CBF-CMRglc in a normal brain at rest (Fox et al., 1988; Lin et al., 2010).

These reduced glucose uptake results led us to hypothesize that in order to sustain essential mitochondrial activity and neuronal functions, the brain may utilize alternative fuel substrates as an energy source. As the brain would also use ketone bodies as energy source (Akram, 2013), we measured ketone body levels in the brain and blood and found that CR rodents had a significantly higher concentration of ketone bodies in comparison to AL animals (Guo et al., 2015; Lin et al., 2015). The findings indicated that CR may, at a very early stage in the brain, induce a metabolic switch from glucose to ketones.

To verify the impact of elevated ketone bodies on vascular functions, we fed young, age-matched WT mice with the KD for 16 weeks. Similar to what we observed with young CR mice, mice fed with KD also had significant increases in CBF and P-gp transport activity levels in brain capillaries compared to control mice (Ma et al., 2018). These neurovascular enhancements were also associated with reduced mTOR and increased eNOS protein expressions. The result is consistent with previous reports that ketogenesis is associated with the down-regulation of mTOR (McDaniel et al., 2011). In line with this, two other studies indicate that an acute increase in ketone body concentration (via infusion of β-hydroxyl butyrate) elevated CBF independent of overall cerebral metabolic activity. This suggests that ketone bodies can directly increase CBF via the cerebral endothelium (Hasselbalch et al., 1996; Roy et al., 2013).

## Caloric Restriction Preserves Brain Vascular and Metabolic Functions in Aging Rodents

Healthy aging is accompanied by CBF reduction, BBB impairment and Aβ retention (Lin et al., 2015; Parikh et al., 2016; Hoffman et al., 2017). To identify CR effects on the aging brain, we included old CR and AL mice (18–20 months of age) in the same CR experiments and compared them with young mice (5–6 months of age). We found that CR enhanced CBF in young mice; moreover, CR also reduced the CBF decline in aging (Parikh et al., 2016). As a result, when compared to young AL mice, old CR mice had comparable levels of CBF, indicating that CR preserves CBF with age. Similar results were found in rats, showing that old rats with chronic CR diet had much higher CBF compared to the age-matched animal, and had comparable CBF level compared to the young AL rats (Lin et al., 2015). These results support that CR has an early enhancement effect on CBF that is preserved with aging. In addition, the preserved CBF in the hippocampus and frontal cortex were highly associated with the preserved memory and learning, as well as the reduced anxiety (Parikh et al., 2016). Our results suggest that dietary intervention initiated at a young age (e.g., young adults) may prove beneficial in the preservation of cognitive and mental abilities in aging.

A similar trend was also observed in hippocampal tCr concentration. As mentioned above, tCr was enhanced in young CR mice. Although tCr dropped dramatically as the CR mice getting older, tCr levels remained comparable to those in young AL mice and were higher than those in old AL mice. This suggests that CR increases ATP production in young CR mice while preserving ATP production in old CR mice (Guo et al., 2015).

Using advanced MRS techniques like POCE, we were able to trace *in vivo* mitochondrial oxidative metabolisms in neurons and neurotransmitter cycling between neuronal and glial cells (Lin et al., 2014). We found that, compared with the young AL rats, old CR rats had similar levels of neuronal glucose oxidation and neurotransmitter cycling, suggesting CR preserved mitochondrial functions and neuronal activity with age. In contrast, old AL rats had much lower levels in both measures. When calculating the ATP production rates for the three groups we found that old CR and young AL animals also had comparable levels of ATP production. We also observed metabolic shifts in aging animals. When compared to age-matched AL rats, old CR rats had significantly lower glucose uptake values in the various brain regions but had significantly higher levels of ketone bodies, β-hydroxyl butyrate (BHB) in the brain (Lin et al., 2015). The metabolic shift may play a critical role in sustaining brain energetics in aging.

Taken together, using multi-modal imaging methods we demonstrated that CR enhances vascular and metabolic functions in early life stages and decelerates the decline with age. Maintaining a healthy brain homeostasis may be due to the metabolic shift from glucose to ketone bodies (Lin et al., 2017b).

## Translational Potential of mTor Interventions in Clinical Trials

Many mTOR inhibitors (including rapamycin, rapalogs, and Everolimus) have already been approved by the FDA and are widely used in clinics (Soefje et al., 2011). Since 1999, rapamycin, alongside other immunosuppressive agents, has been administered to transplant patients to prevent the rejection of organs (Camardo, 2003). Over the past decade, studies also showed that rapamycin or rapalogs have an anti-tumor property; for relatively long periods of time, cancer patients with rapamycin show little change in their quality of life (Mita et al., 2003). Other studies reported that Everolimus improved cognition and reduced depression in humans (Lang et al., 2009). Recent studies showed that with low doses of rapamycin (e.g., lower than half of the therapeutic dosage; 0.5 mg daily or 5 mg weekly), cognitively normal elderly had improved immune functions with minimal side effects (Mannick et al., 2014). The results of the studies support that a short-term rapamycin treatment can be used safely in otherwise healthy older person.

To date, most rapamycin and rapalog clinical studies focus on structural neuroimaging to examine changes in brain tumor mass (Tillema et al., 2012; Fukumura et al., 2015; Ma et al., 2015; Sasongko et al., 2016), metastatic cancer (Subbiah et al., 2015), or active lesions (Moraal et al., 2010). However, functional neuroimaging such as EEG has been clinically applied to assess the efficacy of rapamycin in treating epilepsy (Cambiaghi et al., 2015), and MRS was used to study metabolic implications of rapamycin (Serkova et al., 1999).

CR has also been studied in humans. A recent publication by Redman et al. shows that young, healthy individuals having achieved 15% CR experienced about 8 kg weight loss over 2 years (Redman et al., 2018). Energy expenditure (measured over 24 h of awake and sleep cycle) was reduced beyond weight loss and systemic oxidative stress was also reduced. Findings from this 2 year CR trial in healthy, non-obese humans provide new evidence of persistent systemic metabolic slowing accompanied by reduced oxidative stress, which supports the rate of living and oxidative damage theories of mammalian aging.

CR has also been observed to improve memory in older adults (Fontan-Lozano et al., 2008; Witte et al., 2009; Mattson, 2010; Valdez et al., 2010). Using functional and structural MRI measurements, Witte et al. (2014) reported that resveratrol, a CR-mimetic nutrient, enhanced word retention over a 30-min period in older adults when compared with placebo. These results support that supplementary resveratrol can improve memory performance, as well as improve glucose metabolism and increase hippocampal functional connectivity in older adults. In another study, Jakobsdottir et al. (2016) reported that CR reserved abnormal brain activity in brain areas (e.g., amygdala) involved in the processing of visual food-related stimuli in postmenopausal women with obesity. It should be noted, however, there are studies that these studies have only investigated the short-term benefits of CR.

The KD has been used in the clinic to treat epilepsy (Baranano and Hartman, 2008; Walczyk and Wick, 2017), Parkinson’s disease (Vanitallie et al., 2005), and autism (Evangeliou et al., 2003). The use of neuroimaging in clinical KD studies include EEG and functional MRI to define the extent of dysplasia (Guerrini et al., 2015), and ^1^H-MRS to assess GABAergic activity (Wang et al., 2003) and glucose metabolism (Fujii et al., 2007). Recent studies also investigated the efficacy of ketone utilization in the brain. Using PET, it was found that the cerebral metabolic rate of ketones represents about 33% of the brain’s energy requirements after 4 days on KD (Courchesne-Loyer et al., 2017). POCE studies in human have reported that consumption of ketones (BHB) is predominantly neuronal (Pan et al., 2002). These results support that ketone bodies are an effective alternative fuel substrate in the non-fasted adult human brain.

Collectively, rapamycin, CR, and KD have been widely applied to human studies, which indicates that our work with animal models has the potential to be translated to human studies. To date, little has been reported regarding *in vivo* vascular and metabolic measures in aging and AD with these interventions. The use of quantitative neuroimaging methods (e.g., ^18^FDG-PET, POCE, ^1^H-MRS, MRA, and ASL) would be vital in future use to identify the efficacy of mTOR-related interventions and treatments for protecting brain functions in aging and various AD-related neurodegeneration, including vascular dementia and Down syndrome (Lin et al., 2016).

## Conclusion

**FIGURE 1:**
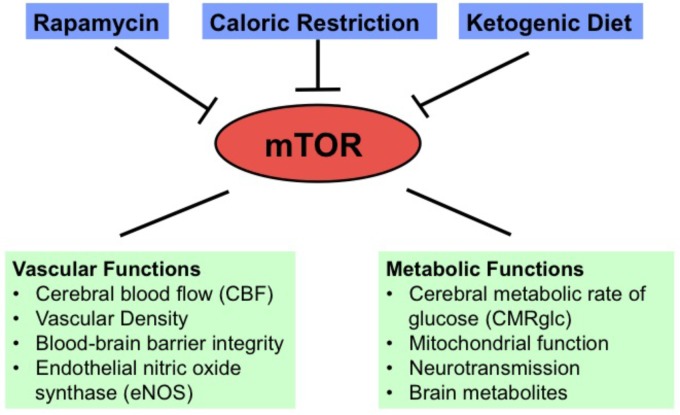
Diagram showing vascular and metabolic changes induced by mTOR inhibition through rapamycin, caloric restriction, and ketogenic diet.

In this review, we discussed the neuroprotective effects of mTOR inhibition in aging and AD. Specifically, rapamycin is a preventative, and possibly a treatment, for the effects of the AD phenotype observed in APOE4 and hAPP(J20) transgenic mouse models of AD; CR and KD can enhance brain vascular functions and shift metabolism in young healthy mice; and CR can preserve brain metabolic and vascular functions in aging. We summarize these findings in **Figure 1**. As the quantitative PET and MRI neuroimaging methods used in these studies in animal models can be translated into human studies, they will be greatly useful in future studies to examine the effects of these mTOR-related interventions in preventing brain function declines associated with aging and neurodegeneration in clinical trials.

## Author Contributions

All authors listed have made a substantial, direct and intellectual contribution to the work, and approved it for publication.

## Conflict of Interest Statement

The authors declare that the research was conducted in the absence of any commercial or financial relationships that could be construed as a potential conflict of interest.
